# An External Patient Healthcare Index (EPHI) for Simulating Spatial Tendencies in Healthcare Seeking Behavior

**DOI:** 10.3389/fpubh.2022.786467

**Published:** 2022-03-31

**Authors:** Jay Pan, Duan Wei, Barnabas C. Seyler, Chao Song, Xiuli Wang

**Affiliations:** ^1^Healthcare Evaluation and Organizational Analysis Group, West China School of Public Health and West China Fourth Hospital, Sichuan University, Chengdu, China; ^2^Institute for Healthy Cities and West China Research Center for Rural Health Development, Sichuan University, Chengdu, China; ^3^People's Government of Jinkouhe District, Leshan, China; ^4^Department of Environment, Sichuan University, Chengdu, China

**Keywords:** EPHI, 2SFCA, healthcare seeking behavior, healthcare resource allocation, efficiency

## Abstract

**Background:**

Healthcare resources are always more limited compared with demand, but better matching supply with demand can improve overall resource efficiency. In countries like China where patients are free to choose healthcare facilities, over-utilization and under-utilization of healthcare resources co-exist because of unreasonable healthcare seeking behavior. However, scholarship regarding the spatial distribution of utilization for healthcare resources, resulting from unreasonable spatial tendencies in healthcare seeking, is rare.

**Methods:**

In this article, we propose a new External Patient Healthcare Index (EPHI) to simulate the spatial distribution of utilization for healthcare resources, based on the Two-Step Floating Catchment Area (2SFCA) method, which is widely used to assess potential spatial accessibility. Instead of using individual-level healthcare utilization data which is difficult to obtain, the EPHI uses institution-level aggregated data, including numbers of inpatient/outpatient visits. By comparing the estimated utilization (based on local healthcare institution services provision) with the expected utilization (based on local population morbidity), guest patients (e.g., patients flowing in for treatment) and bypass patients (patients flowing out) can be identified. To test the applicability of this index, a case study was carried out on China's Hainan Island. The spatial tendencies of patients for inpatient and outpatient services were simulated, then incorporated with spatial access to healthcare resources to evaluate overall resource allocation efficiency, thus guiding future resource allocations and investment for policy makers and healthcare providers.

**Results:**

The EPHI revealed that bypass activities widely exist on Hainan Island in both inpatient and outpatient care, with patients tending to travel from less developed regions with fewer healthcare resources to more highly developed regions with more healthcare resources to receive healthcare. Comparison with spatial accessibility demonstrated how bypass activities on Hainan produced an under-utilization of doctors in less developed regions and over-utilization of doctors in more developed coastal regions.

**Conclusions:**

This case study on Hainan Island demonstrates that this new index can very clearly identify both the sources and sinks of patient spatial tendencies. Combining these results with spatial accessibility of healthcare resources, how efficiently the available supply matches the utilization can be revealed, indicating wide-ranging applicability for local governments and policymakers.

## Introduction

The enjoyment of the highest attainable standard of health without distinction of any kind is a fundamental right of every human being (1). However, no healthcare system can provide unlimited healthcare resources to every user (2). Healthcare resources are always limited compared to demand (reflected by patients), so all healthcare systems, regardless of their organizational structures or financing options, must necessarily employ rationing mechanisms to prioritize finite healthcare resources across their consumers bases (3). In modern society with increasing healthcare burdens, such as aging populations and growing prevalence of chronic non-communicable diseases (4, 5), how to most efficiently allocate limited healthcare resources is gaining increasing attention.

The idealized way of allocating resources is to match available supply with demand. From the supply side, as the largest developing country, serving one fifth of the global population, China has made significant investments in its healthcare system, especially after launching major healthcare reforms in 2009 (4, 6). Provider-to-population ratios (PPRs) were calculated within each administrative boundary to identify resource shortage areas (7, 8). However, disparities within administrative boundaries and cross-boundary healthcare seeking behaviors were not considered (9). With the development of modern geospatial techniques and big data analytical approaches, methods have been utilized to more accurately assess the distribution pattern of healthcare resources, among which the Two-Step Floating Catchment Area (2SFCA) method and its family of variations have gained the most attention (10–13). Studies utilizing the 2SFCA method and its variants to assess healthcare resource allocation in China have been increasing in recent years (14–17). Apart from assessing healthcare resource allocation in different study areas (18–21), modifications on the 2SFCA method have been made to better fit the reality in China, including the utilization of online navigation systems to calculate real time travel impedance to get more accurate spatial access estimates (22, 23), and considering different tiers and/or types of healthcare institutions as well as their catchment area sizes depend on individual research questions (16, 24). Creatively, Zhang et al. (25) and Wang et al. (13) identified different types of healthcare resource shortage areas by overlapping the service catchments of different tiers/types of healthcare institutions.

From the demand side, research regarding the actual distribution of demand for healthcare resources is rare in China, although cross-region healthcare seeking behaviors, namely 跨区域就医 (kuà quyù jiùyi) or 异地就医 (yìdì jiùyi) (referred to as unreasonable healthcare seeking behavior in the following context), are of great concern of the Chinese government. In most studies, the total population is regarded equally as the healthcare demand (12, 13, 16). Some recent studies have calculated healthcare demand for the elderly population separately because they usually have more needs for healthcare services (15, 26). However, one of the fundamental problems with this approach relates to China's socio-cultural focus on healthcare planning. That patients do not actually utilize the healthcare resource that they are expected to has not been fully addressed in current scholarship. In China, people are free to choose healthcare facilities without restriction from gatekeeping mechanisms (27). As a result, bypass activity, which is a type of spatial tendency in healthcare seeking behavior of patients, is quite common in China. Essentially, patients “bypass” the healthcare resources planned for their utilization and instead travel long distances to compete for the healthcare resources that are planned to serve another group or population. The healthcare resources being bypassed are usually perceived as lower in quality and the healthcare resources patients bypass to are usually higher in reputation (27), and resulting in both over-utilization and under-utilization of healthcare resources. This is a remaining challenge associated with the market reforms to China's healthcare system beginning in the early 1980s (28–30), and recent policies have sought to curtail or otherwise constrain bypass activities (31).

Generally, the patterns of spatial tendencies in healthcare seeking can be derived in two ways. The first is from personal medical records, such as from Medicare in the United States (32) or inpatient discharge data in China (33), which includes each patient's residential address and the targeted healthcare institution. Theoretically, personal medical records are most suitable for analyzing the spatial tendencies in healthcare seeking behavior of patients, but, due to privacy concerns, the data are not often easily accessible to researchers (34). Moreover, in China, due to inconsistent quality in record-keeping, there is often considerable data missing from these datasets (33). The second is from interviews and questionnaires, which is more commonly used in China (27). But in these types of studies, the research instruments tend to broadly collect information on the type of healthcare institution patients choose (rather than the exact facility or address) and on general motivating factors influencing patient choices, thereby limiting the usefulness of these data in studies seeking to analyze the spatial relationship between patients and healthcare institutions (35, 36).

Considering the constraints of personal medical records, Delamater et al. (34) recently evaluated the potential utility of floating catchment area metrics for estimating the probability of the people at a residential location to use certain healthcare institutions (predicting spatial patterns of healthcare utilization). They noted that in the 2SFCA method's second step (see Section Measuring Spatial Access: The 2SFCA Method for detail), at each residential location, the supply to demand ratios of the facilities falling within the threshold distance were summed to calculate the final spatial access value at the residential location. The partial access provided by each facility for a residential location can also be considered as describing the probability that consumers living at this residential location will visit a certain facility. By integrating the probability values with the demand size (usually population) at each residential location, a matrix representing the number of consumers in each residential location to all possible healthcare facilities can be generated. In their case study in Michigan, Delamater et al. (34) used hospital beds as the parameter representing a healthcare institution's “attractiveness”, finding that the percent of correctly predicted visits reached as high as 70%. This suggests that the 2SFCA metrics have the potential to estimate where people go to receive healthcare when detailed utilization data is lacking. Applying Delamater et al.'s methods to the whole of Germany, Bauer et al. (37) demonstrated that using actual hospital visits rather than hospital beds is a more accurate way of predicting spatial patterns of healthcare utilization.

Building upon the insights provided by these previous studies, and in light of the urgent requirement to correctly measure healthcare utilization to more efficiently allocate healthcare resources in China, we propose a new index called the External Patient Healthcare Index (EPHI) to estimate the spatial distribution of healthcare utilization. Rather than culminating in a difficult-to interpret matrix to represent the relationship between consumers at each residential location and all possible healthcare facilities, our method more clearly/simply identifies patient flow-in (utilization is larger than demand) and patient flow-out areas (utilization is smaller than demand) to represent the spatial tendencies in healthcare seeking behavior of patients. We take South China's Hainan Island as a case study location to test the utility of the EPHI index. The estimated spatial distribution of healthcare utilization identified by this index were compared to Hainan's current resource allocation plan to assess the actual vs. idealized efficiency of resource allocation. This allows clear recommendations to be proposed for the local government on how to optimize healthcare resource allocation, including the allocation of healthcare resources in existing healthcare institutions and the investment of new healthcare institutions or enhancement of existing primary healthcare institutions.

## Materials and Methods

### Measuring Spatial Access: The 2SFCA Method

Spatial access (short for potential spatial access) represents the geographical convenience in obtaining services (12, 38). The 2SFCA method, first formally proposed in 2003 by Luo and Wang (39), has gained the most attention in measuring spatial accessibility, especially in its application to healthcare access (12, 13). Based on the gravity model, the 2SFCA method emphasizes the importance of spatial separation between supply and demand and how they are connected in space (38). However, in contrast with the gravity model, the 2SFCA method output is provided in an easy-to-interpret supply to population ratio (40). The 2SFCA method offers substantial theoretical advantages over traditional container-based regional availability measures (e.g., PPRs). Shortcomings identified with the container-based methods include that they: (1) cannot reveal detailed spatial variations within large service areas, and (2) assume that boundaries are impermeable, meaning that actual interactions across boundaries are not adequately considered (39). These shortcomings are overcome by allowing containers to “float” as catchments or travel buffers based on travel time or distance from the facility and population locations.

In the 2SFCA method, the supply point *j* belongs to a set of supply points {1, 2, …*n*}, demand point *i* belongs to a set of demand points {1, 2, …*m*}, the travel impedance (distance or travel time) between supply point *j* and demand point *i* is *d*_*ij*_. The service capacity (e.g., hospital beds), sometimes also recognized as the “attractiveness” of supply point *j*, is *S*_*j*_. The demand size of demand point *i*, usually the population, is *P*_*i*_. The threshold travel impedance, which is the longest travel time/largest travel distance demanders will travel, is *D*_*o*_.

In the first step, floating catchments are generated as centralizing supply points. For each supply point *j*, all demand points (*i*) are searched within the threshold travel impedance (*D*_*o*_) from the supply point (*j*), which is the floating catchment area of point *j*. The supply-to-demand ratio (*R*_*j*_) is then computed within the floating catchment area.


(1) $$\begin{array}{l} R_{j}=\frac{S_{j}}{\sum_{i\in \left\{d_{ij}\leq D_{0}\right\}}P_{k}} \end{array}$$

In the second step, floating catchments are generated as centralizing demand points. For each demand point *i*, all supply points (*j*) are searched within the threshold travel impedance (*D*_*o*_) from the demand point (*i*), services provided by these suppliers are assumed to be accessible at demand point *i*. The supply-to-demand ratios (*R*_*j*_) are summed up to get the accessibility (*A*_*i*_) at demand point *i*.


(2) $$\begin{array}{l} A_{i}=\underset{j\in \left\{d_{ij}\leq D_{0}\right\}}{\sum }R_{j} \end{array}$$

The 2SFCA method essentially produces an “accessibility score” representing the ratio of supply to demand, with each interacting together in a distance decay trend (41). That is, it represents the convenience that residents from one location have in reaching and obtaining services from multiple possible facilities. Consequently, the larger *A*_*i*_ value indicates better access at location *i*. Since its inception, the 2SFCA method quickly became a popular spatial accessibility measure (41), with most of the recent 2SFCA-related research focusing on using it to identify and map disparities in healthcare accessibility (10–13), or proposing methodological improvements to the metrics. Multiple improvements have been proposed based on the 2SFCA (13, 16) and the general goal of these methods are to more accurately model service seeking behaviors.

The 2SFCA method assumes equal accessibility within catchments but no outside catchment accessibility, so the Enhanced Two Step Floating Catchment Area (E2SFCA) method was developed as a significant advance to overcome these limitations (13, 42). Compared with 2SFCA, the E2SFCA method first divides each catchment into several sub-catchments, then applies various weights for each sub-catchment in order to represent the distance decay trend (42). A weight function, which can be modified depending on the type or importance of a service or resource, is used to define the sub-catchment weights (13, 43). Due to its methodological rigor and versatile applications, the E2SFCA method has been used in various regions and socio-cultural contexts around the world, including in China (12, 13, 18, 44–47). The E2SFCA method can be represented as follows:


(3) $$\begin{array}{l} R_{j}=\frac{S_{j}}{\sum_{i\in \left(d_{ij}\in D_{0}\right)}P_{i}f\left(d_{ij}\right)}=\frac{S_{j}}{\sum_{i\in \left(d_{ij}\in D_{r}\right)}P_{i}W_{ij}} \end{array}$$

(4) $$\begin{array}{l} A_{i}^{F}=\underset{j\in \left(d_{ij}\in D_{0}\right)}{\sum }R_{j}f\left(d_{ij}\right)=\underset{j\in \left(d_{ij}\in D_{r}\right)}{\sum }R_{j}W_{ij} \end{array}$$

The threshold travel impedance *D*_*o*_ is split into multiple sections (*r* sections), and *D*_*r*_ is the *r*^*th*^ service area ring from the central location. As the service area ring gets further form the center, the possibility that the supply interacts with the demand will decrease. The distance-weights (*W*_*ij*_) variable in the formula was theoretically specific for each combination of supply point *j* and demand point *i* and represents the decreasing likelihood of utilizing healthcare resources at greater distances. In the E2SFCA method, the possibility that the supply interacts with the demand is assumed to be the same within each sub-catchment (service area ring), so for all *d*_*ij*_ within the range of *D*_*r*_ the distance-weight *W*_*ij*_ can be simplified to *W*_*r*_.

### Measuring Spatial Distribution of Utilization for Healthcare Resources: The External Patient Healthcare Index

Aiming at revealing the spatial distribution of utilization for healthcare resources, which is resulted from unreasonable spatial tendencies in healthcare seeking behavior, an EPHI was proposed as follow:


(5) $$\begin{array}{l} EPHI_{i}=\frac{{\text{Estimated Utilization}}_{i}}{{\text{Expected Utilization}}_{i}} \end{array}$$

For each residential point *i*, the EPHI value is calculated by dividing the *estimated utilization* with the *expected utilization*. The expected utilization, which is defined as the theoretical number of patients at residential point *i*, can be understood as the potential demand at residential point *i*, and was calculated based on local population morbidity in this study. The estimated utilization, which is defined as the actual number of patients at residential point *i*, can be understood as the actual utilization at residential point *i*, and was calculated based on local healthcare institution services provision in this study.

The EPHI value can be understood from two aspects. Under the assumption that patients can only get services locally, a larger EPHI value indicates that population utilizes healthcare services more frequently than anticipated and the healthcare resources are over-utilized by local residents. Meanwhile, a smaller EPHI value indicates that population utilizes healthcare services less frequently than anticipated and the healthcare resources are under-utilized by local residents. Under the assumption that patients are free to seek for healthcare resources everywhere, which can be understood as the spatial tendencies in healthcare seeking, the explanation of EPHI values will be different. A larger EPHI value indicates that the local healthcare utilization is higher than the anticipated healthcare demand from local residents, and this represents patients outside the residential point *i* (i.e., *guest patients*) flowing into the area for healthcare. Essentially, the larger the EPHI value is, the higher the utilization of services is (i.e., more guest patients). In contrast, a smaller EPHI value indicates that the local healthcare utilization is less than the anticipated healthcare needs of local residents, indicating local patients flow out for healthcare (i.e., *bypass patients*). The smaller EPHI value is, the more bypass patients there are. In the real world, the EPHI value should be the combination of both, while in this study, with the purpose of identifying unreasonable spatial tendencies in healthcare seeking behavior (which is common and troublesome to the government), an emphasis was put on the second assumption that patients are free to seek for healthcare resources everywhere.

Inspired by Delamater et al. (34), the *estimate utilization* in the EPHI was calculated based on the theoretical framework of the 2SFCA method, considering the widespread utilization and advantages of the E2SFCA method, a distance decay weight was also introduces. Theoretically, in the E2SFCA method, for the first step, healthcare resources within a healthcare institution are treated as being shared by the population within the catchment of the institution. And for the second step, the services provided by accessible healthcare institutions are summed up to obtain each residential location's accessibility. From another perspective, the patients going to a healthcare institution (utilizing certain healthcare institution's resource) are essentially “contributed” by the population within the catchment of the healthcare institution. The patients from residential locations going to different healthcare institutions can be summed to get the total number of patients from each residential location. Thus, the *estimate utilization* for each residential point *i* can be described as follows.

In the first step, the utilization of care from each specific healthcare institution *j* is assigned to neighboring residential points based on a distance decay function within the service area. The *Utilization-Population Ratio* (*U*_*j*_) for healthcare institution (indicating the average utilization for every resident located within the service catchment area of institution *j*) is calculated using the following formula:


(6) $$\begin{array}{l} U_{j}=\frac{V_{j}}{\sum_{i\in \left(d_{ij}\in D_{0}\right)}P_{i}f\left(d_{ij}\right)}=\frac{V_{j}}{\sum_{i\in \left(d_{ij}\in D_{r}\right)}P_{i}W_{ij}} \end{array}$$

where *U*_*j*_ is the *Utilization-Population Ratio* for institution *j*. *V*_*j*_ is the volume of healthcare provision (corresponding to patient utilization) of healthcare institution *j*. *D*_*r*_ is the *r*^*th*^ service area ring of institution *j*, *d*_*ij*_ is the distance between a residential point *i* (within the service area ring of institution *j*) and the location of *j*, *P*_*i*_ is the population at residential point *i*, and *W*_*ij*_ is the distance decay weight for residential point *i* and institution *j*, during the calculation, *W*_*ij*_ is the same within service area ring *D*_*r*_.

In the second step, the total healthcare utilization number at each residential point *i* (*estimate utilization* for residential point *i*) is calculated by summing the utilization of each reachable institution *j*:


(7) $$\begin{array}{l} {\text{Estimated Utilization}}_{i}=\underset{j\in \left(d_{ij}\in D_{0}\right)}{\sum }{P_{i}U}_{j}f\left(d_{ij}\right)\\ =\underset{j\in \left(d_{ij}\in D_{r}\right)}{\sum }P_{i}U_{j}W_{ij} \end{array}$$

In this formula, *U*_*j*_ is the Utilization-Population Ratio indicating the average utilization for every resident located within the service catchment area of institution *j*. *D*_*r*_ is the “seeking” catchment area of residential point *i*, *d*_*ij*_ is the distance between residential point *i* and institution *j*, and *W*_*ij*_ is the distance decay weight for residential point *i* and institution *j*, during the calculation, *W*_*ij*_ is the same within service area ring *D*_*r*_.

The *expected utilization* was calculated based on local population morbidity in this study. Due to data limitation, the *expected utilization* for each residential point *i* in the study area was considered to be the same, while the population morbidity would be different based on social, demographic, and economic reasons. The *expected utilization* for residential point *i* is calculated using the following formula:


(8) $${\text{Expected Utilization}}_{i}=(\frac{\sum V_{j}}{\sum P_{i}})P_{i}$$

where *V*_*j*_ is the volume of healthcare provision (corresponding to patient utilization) of healthcare institution *j*, *P*_*i*_ is the population at residential point *i*.

For data preparation of the EPHI calculation, apart from the demand and supply locations, as well as travel costs between them, the institution-level aggregated data (e.g., number of inpatient and outpatient visits), which are easier to obtain and more accurate than personal medical records in China's healthcare information system, are collected from yearly reports of healthcare institutions.

### Study Location

Located just off the coast of southern China, Hainan Island was chosen to apply the methods proposed in this study because the large-scale cross-boundary health seeking behaviors typical of patients in other areas of China is relatively limited on Hainan (13). This is because, despite its close proximity to Mainland China, as an island, Hainan functions as a relatively closed-loop system ideal for analyses of the impact of healthcare policies and resource allocation efficiency (13). Furthermore, unlike the more economically developed cities along China's southern and eastern coastlines, such as Guangzhou, Shenzhen, and Shanghai, which are very dynamic and have previously implemented unique healthcare reforms, Hainan's provincial government tends to strictly follow policy directives from China's Central Government in Beijing. Consequently, the findings of studies on Hainan Island have great potential application far beyond the island itself (13).

**Figure 1:**
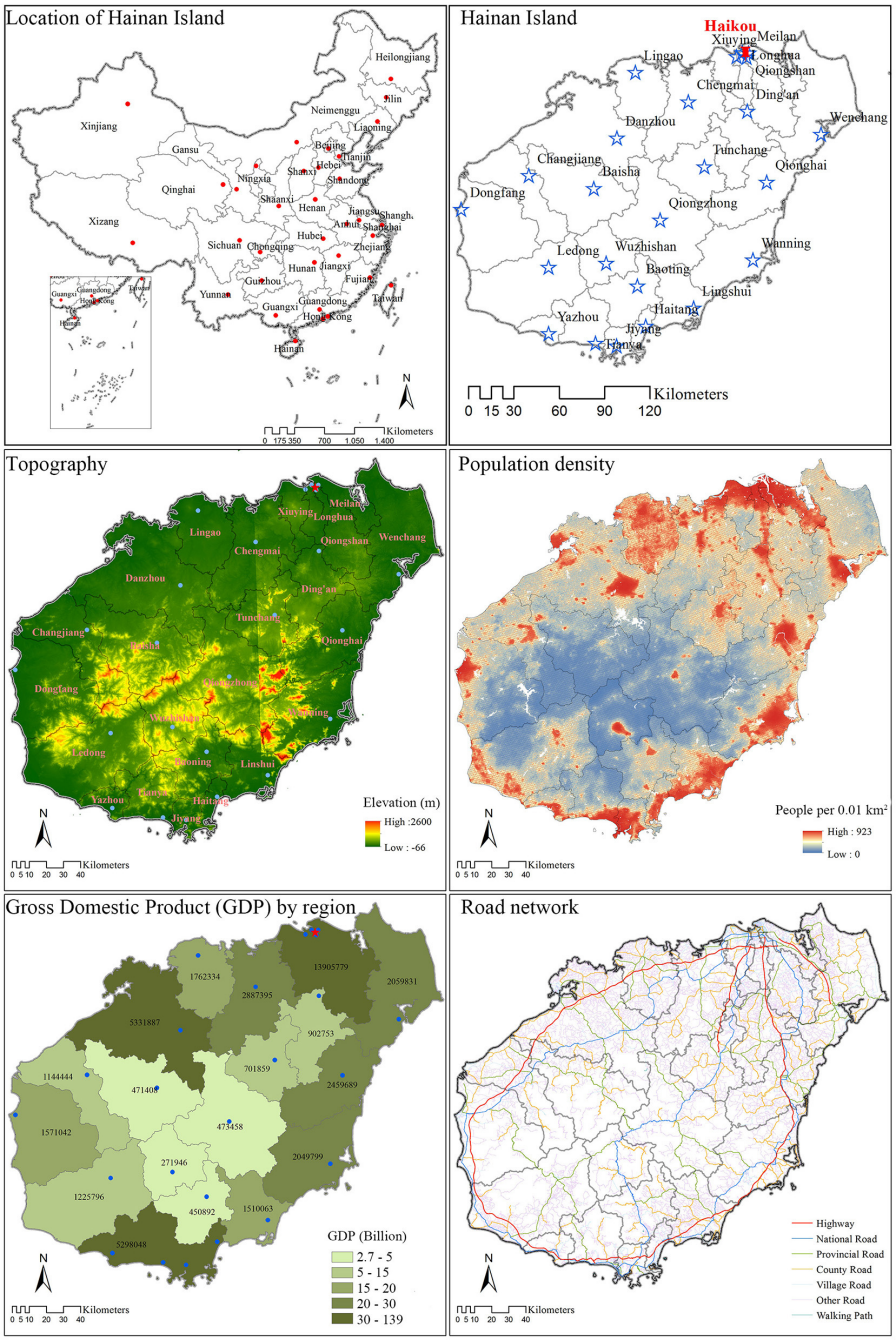
Study area population, topography, transportation, and economic development on Hainan Island, China (2018). Distribution of study area, administrative boundaries, county centers, topography (elevation), and road network were downloaded from the National Geomatics Center of China. The Gross Domestic Product (GDP) by region indication economic development was derived from Hainan Statistical Yearbook 2019. The population density to grid level (100*100 m^2^) was downloaded WorldPop.

Hainan Island's total area is about 34,000 km^2^, and its total population (2018 permanent population) is 9.34 million (48). Due to its rugged topography and mountainous central area, the coastal regions on Hainan Island are much more developed, in terms of roads and other infrastructure, but also with greater gross regional products. The island's population is also distributed much more densely along the coasts than the center of the island. That is to say, Hainan's central region is the most mountainous, is the least developed, and has the lowest population density (13) (Figure 1).

### Data Sources and Pretreatment

This study was designed to simulate the spatial tendency for healthcare seeking behavior with reference to spatial accessibility methods, thus three types of data were required: (1) demand-side data, (2) supply-side data, and (3) geographical impedance between suppliers and demanders (49).

The supply side data were provided by the Health Bureau of Hainan Province. For every healthcare institution, general data (e.g., name, address, etc.), service capacity [e.g., numbers of beds and registered (assistant) doctors], and utilization data (e.g., number of inpatient/outpatient visits) for 2018 were extracted from the yearly report system. All healthcare institutions were geocoded by address using Baidu Map, and then saved as points in ArcGIS 10.5 with service capacity and utilization data being attributes.

Since no specific disease was considered in this study, the total population was regarded as potential healthcare demanders. We utilized population data from two sources: (1) WorldPop, which provided estimates of population per (100^*^100 m^2^) grid with national totals adjusted to correspond with UN Department of Economic and Social Affairs' Population Division estimates from 2010, 2015, and 2020 (50), and (2) the Hainan Statistical Yearbook 2019 (48), which provided detailed population data for each district/county in 2018. We regarded the statistical yearbook data as real population data, allocating them to (100^*^100 m^2^) grids according to the WorldPop dataset value per cell, thereby forming a more detailed and precise population distribution map, with the following formula:


(9) $$\begin{array}{l} P_{ic}=G_{ic}^{*}\left(\frac{T_{c}}{G_{c}}\right) \end{array}$$

where *P*_*ic*_ is the corrected population density value of grid *i* in the district/county *c*, *G*_*ic*_ is the corresponding population density value from WorldPop. *G*_*c*_ is the sum of the WorldPop grid values for district/county *c*, and *T*_*c*_ is the real population from the Hainan Statistical Yearbook 2019 in district/county *c*. Each grid represents a population point and the value of the grid was set as the population number at that location.

For this study, geographical impedance was represented by travel time along the road network. The road network data were downloaded from the National Geomatics Center of China (51). For each road section, speed limits were assigned based on road type and speed class, with the slowest being 5 km/h and the fastest being 90 km/h. Other supportive data (e.g., administrative boundaries and elevation data) were also downloaded from the National Geomatics Center of China.

### Procedures

The EPHI values were firstly calculated. The inpatient visits and outpatient visits were calculated separately on Hainan Island to reveal their distinct spatial tendency patterns. All healthcare institutions with inpatient/outpatient records were considered service providers, and the total population was treated as potential demanders. Due to the lack of more detailed data, the expected utilization by all residents for inpatient/outpatient services was considered even across Hainan Island, and calculated by dividing total inpatient/outpatient visits by the total population on Hainan Island in 2018.

The efficiency of healthcare resources was then assessed. In 2012 Shi et al. (52) evaluated the potentially unfulfilled demand (insufficiency) of cancer care in the Unites States by dividing the potential cancer patients (calculated by multiplying population by cancer rate) by the potential spatial access to cancer care, which can be explained as the poorer the spatial access and the more patients at a location, the higher the demand for the service. In this study we similarly calculated the ratio between the estimated utilization (number of patients actually utilized healthcare resource allocated to the residential point) and the potential spatial access to healthcare resources (number of healthcare resources allocated to the residential point to serve the local patients). The higher the ratio is, the more patients are actually served by per unit of resource, in other words, the resource is utilized more efficiently. The insufficiency concept was not suitable in this study because the utilization of healthcare resources is resulted from unreasonable healthcare seeking behavior and is not expected by the government and policy makers, while “receiving treatment locally” and improve the efficiency of healthcare resource are the political goals. In this study, the E2SFCA method was utilized to assess spatial access to beds and doctors (both registered assistant doctors and registered doctors). The estimated utilization of healthcare resources (actual utilization) was then overlapped with the spatial access to identify the efficiency of current resource allocation. Considering beds are a critical resource when inpatient visits occur, and doctors are a critical resource when outpatient visits occur, the estimated utilization of inpatient visits was overlapped with the spatial access to beds, and the estimated utilization of outpatient visits was overlapped with the spatial access to doctors.

The calculation was implemented in ArcGIS 10.5. To be consistent with previous research (13), for each resident point and healthcare institution three catchment areas were generated (0–15, 15–30, 30–60 min). Using the Gaussian function with β being 440, weights for the three catchment areas were calculated to be 0.880, 0.316, and 0.01, respectively.

## Results

### General Description

In 2018, there were 255 hospitals and 4,979 primary healthcare institutions on Hainan Island. The latter includes community health centers/stations, township health centers, clinics, and village clinics. Hospitals were limited in number but control the majority of healthcare resources. In total, there were 12,737 registered doctors, 1,173 registered assistant doctors, and 44,627 beds in hospitals. Although primary healthcare institutions were much larger in number, the healthcare resources they controlled were very limited. In total, among primary healthcare institutions there were only 5,335 registered doctors, 2,268 registered assistant doctors, and 7,039 beds.

**Figure 2:**
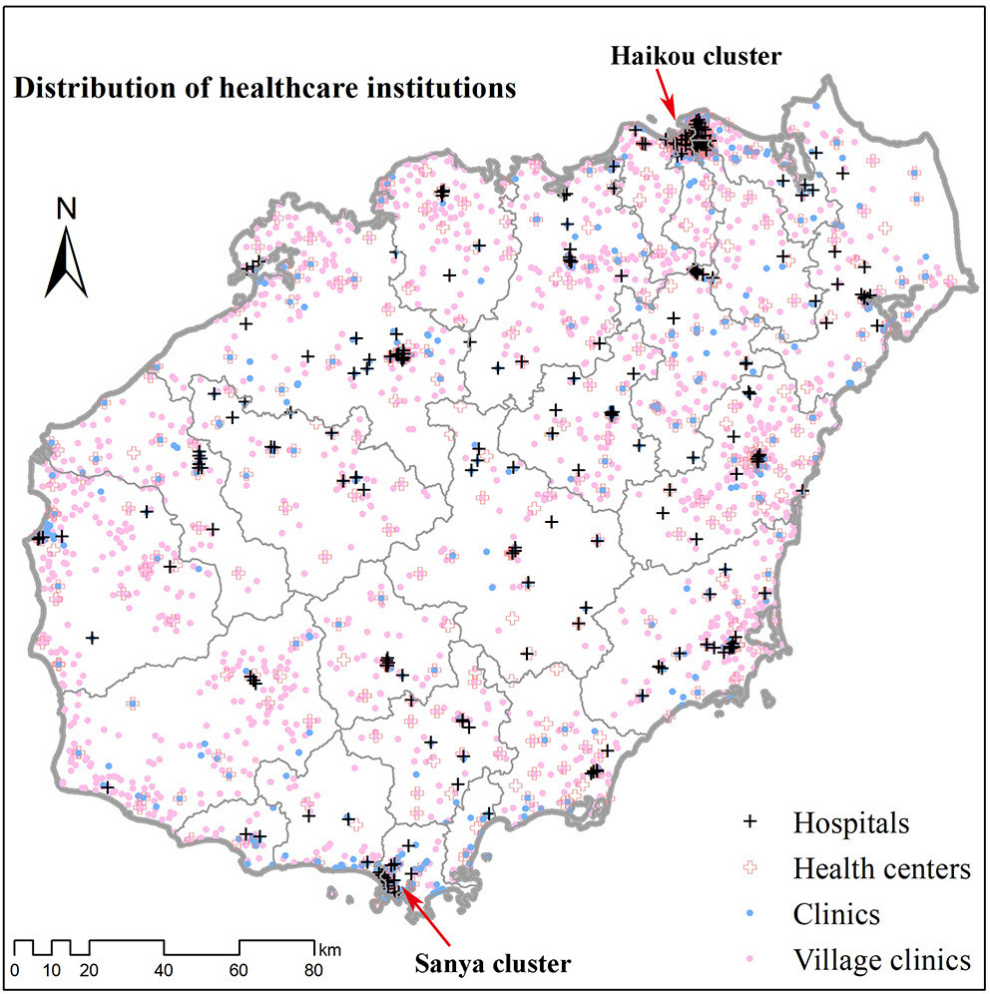
Distribution of healthcare institutions on Hainan Island (2018). Healthcare institution data including hospitals, health centers, clinics, and village clinics were provided by the Health Bureau of Hainan Province. All healthcare institutions were geocoded by address using Baidu Map.

In terms of geographic distribution, Hainan's healthcare institutions were widely distributed across the island. Hospitals tended to be located near the district/county seats, but were mostly concentrated in the island's capital, Haikou City, and the largest tourist beach city, Sanya City (Figure 2). Primary healthcare institutions were also clustered near district/county seats, but were more evenly distributed compared with hospitals. Nonetheless, the most significant primary healthcare institution cluster was also located in Haikou.

In 2018, there were 50,758,044 outpatient visits, and 1,191,184 inpatient visits on Hainan Island. On average, there were 5,459 outpatient and 128 inpatient visits for every 1,000 people. Hospitals provided more than 45% of the outpatient visits, but more than 92% of the inpatient visits. Compared to the national average, the per capital times of outpatient and inpatient visits on Hainan Island were both lower, the percentage of outpatient visits served by hospitals was slightly higher, while the percentage of inpatient visits served by hospitals was much higher.

### Spatial Tendency for Healthcare Seeking Behavior

**Figure 3:**
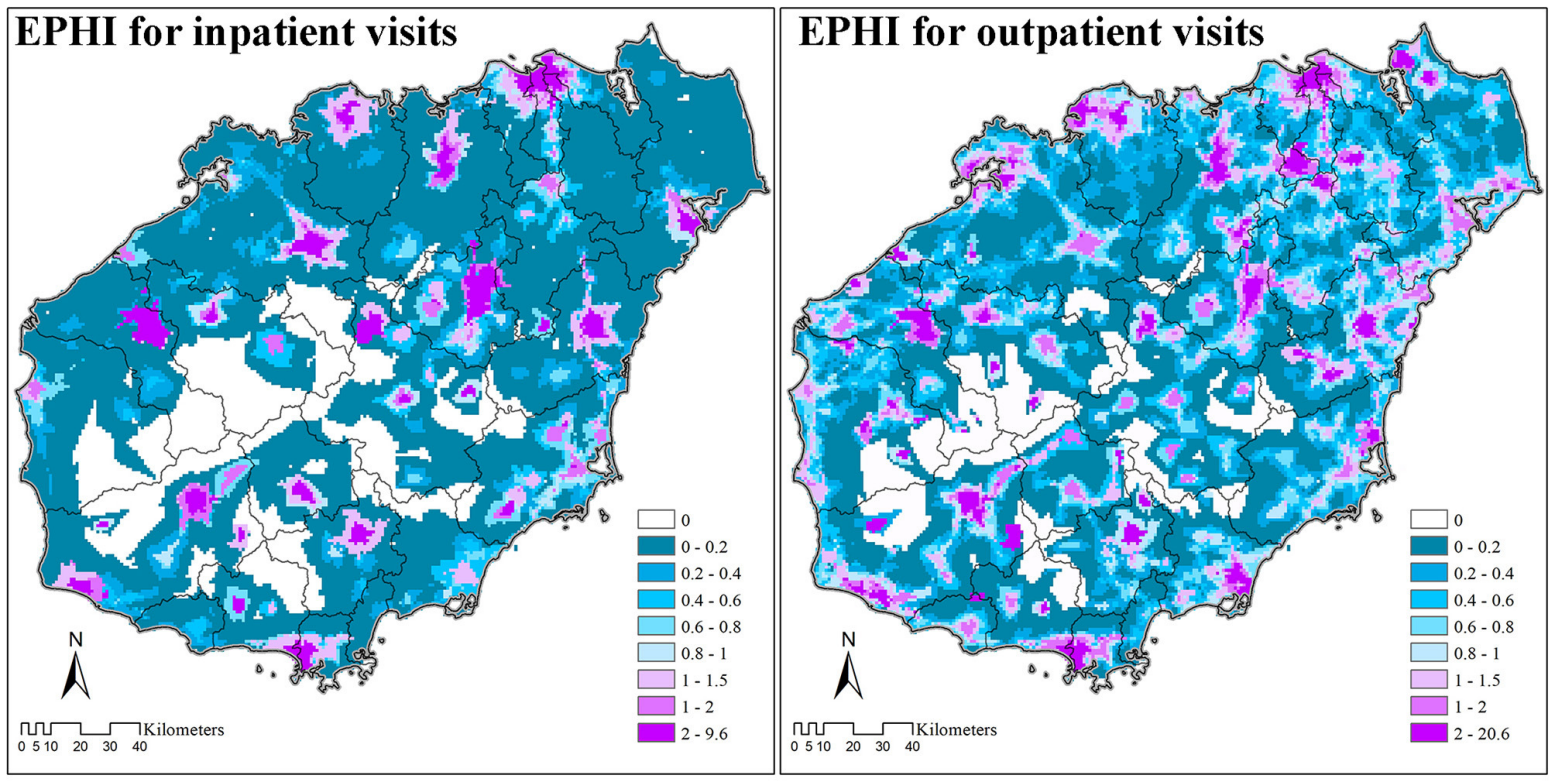
External Patient Healthcare Index (EPHI) of inpatient and outpatient visits on Hainan Island (2018).

For inpatient needs, most patients on Hainan Island travel to hospitals for service (Table 1; Figure 3). Consequently, most areas on Hainan Island are patient flow-out areas, with destinations (sinks) mostly being clustered around hospitals (near administrative centers of districts/counties). The EPHI values are usually quite low (lower than 0.2), indicating that a large proportion of local patients travel outside of their expected utilization catchment areas to get inpatient services (i.e., exhibiting bypass behaviors).

**Table 1:** Comparison of healthcare utilization on Hainan Island and China.

		**Hainan Island**	**Mainland China (including Hainan)**
Outpatient visits	Total (person-visits in millions)	50.76	8,308.02
	Per capital times of outpatient visits	5.46	5.96
	Percentage provided by hospitals (%)	45.64	43.06
Inpatient visits	Total (person-visits in millions)	1.19	254.53
	Per capital times of inpatient visits	0.13	0.18
	Percentage provided by hospitals (%)	92.52	78.64

*Data of mainland China was extracted from Health Statistics Yearbook of China 2019, data of Hainan Island was calculated based on the reported number of outpatient/ inpatient visits of every healthcare institution on Hainan Island and population data extracted from Hainan Statistical Yearbook 2019*.

For outpatient requirements, the distribution of EPHI values is more even. There are more flow-in areas, indicating patients have more choices. The general EPHI values are also higher compared with inpatient visits, indicating even in flow-out areas that there are fewer flow-out patients. However, the maximum EPHI value for outpatient visits is larger than inpatient visits, indicating the burden in some flow-in areas is larger because considerable numbers of guest patients cluster in these areas to acquire local healthcare service.

### Spatial Access to Healthcare Resources

**Figure 4:**
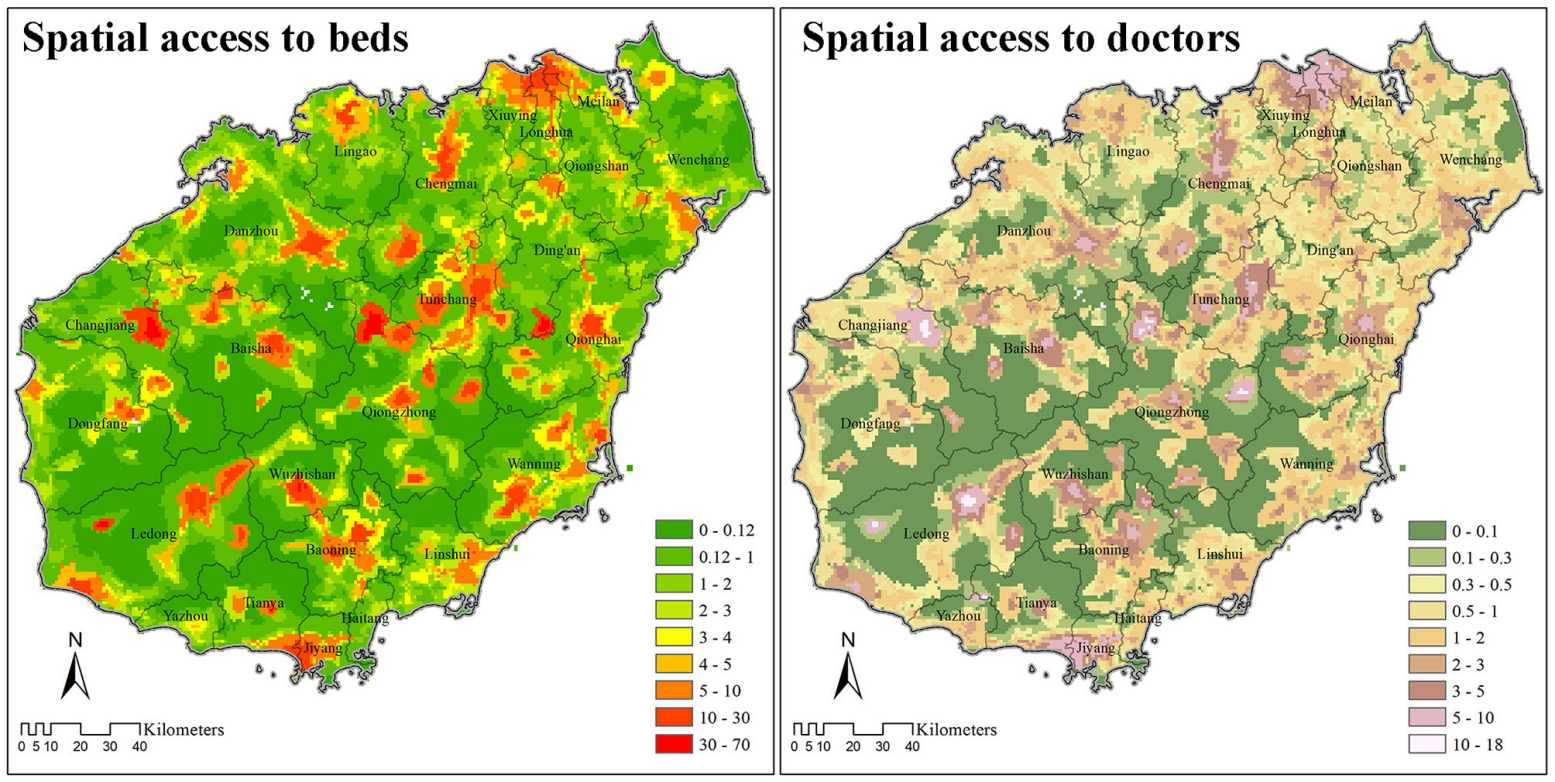
Spatial access to beds and doctors (including registered and registered-assistant doctors) based on E2SFCA method on Hainan Island (2018).

The weighted average of spatial access to beds was 4.81 in 2018, with 84.20% (4.05) being provided by hospitals. Meanwhile, the weighted average of spatial access to doctors was 2.32, with 64.66% (1.50) provided by hospitals. The distribution pattern of spatial access to beds and doctors was similar, but spatial access to beds was more clustered compared with access to doctors (Figure 4). In general, spatial access to healthcare resources (e.g., beds, doctors) was more even along the coasts and the northeast part of Hainan Island, but more divided in the central mountainous areas.

### Efficiency of Healthcare Resources

The estimated number of inpatient visits was divided by spatial access to beds to reveal the competition of beds. In most areas, one bed was utilized by no less than ten patients per year. The few areas where the supply of beds was more sufficient than the demand were mostly located in the central mountainous areas, and the northeast tip of Hainan Island.

Similarly, the estimated number of outpatient visits divided by spatial access to doctors revealed the competition for doctors on Hainan Island. The competition for doctors was less severe compared with that for beds, especially in the central mountainous area, where one doctor serves <2,000 patients every year. In general, the supply of doctors was more efficient along the coastal areas, except for the two major urban areas of Haikou and Sanya.

## Discussion

In this study, we proposed an EPHI to estimate the spatial distribution of healthcare utilization, which can be utilized as a third way of simulate the spatial tendencies for healthcare seeking behavior of patients (27, 32). The index has wide practical application since the data it requires can be obtained more easily compared with personal medical records (34), and the spatial relationship between patients and healthcare institutions can be revealed more clearly than with interviews and questionnaires (27). In comparison with Delamater et al.'s prior research which utilized the idea of 2SFCA method to obtain a matrix representing the number of consumers in each residential location to all possible healthcare facilities (34), this index more directly identifies the sources and sinks of patient spatial movements. As “receiving treatment locally” is a priority of many local governments and increasingly mentioned in healthcare policies (31), this index has wide applicability for assessing the efficiency of local healthcare systems in determining whether patients are seeking healthcare locally. The current indicator for “receiving treatment locally” is “percent receiving treatment within a county” (县域内就诊率, xiànyù nèi jiùzhěn lu), but the detailed data required and calculation methods have not been clearly described, and the results are restricted to county level administrative units (53).

In addition, combined with assessments of spatial access to healthcare resources, the competition for healthcare resources (e.g., the efficiency of supply vs. utilization) can be straightforwardly assessed with the index for every residential point. From the “people oriented” (以人为本, yirénwéiběn) perspective, which is one of the central tenets of Chinese policy making (54), the convenience of patients receiving healthcare services is of great importance. However, existing literature usually assesses efficiencies of healthcare resources based on administrative units (55), or healthcare institutions, because the invested resources and the outputs are easy to obtain (56, 57).

**Figure 5:**
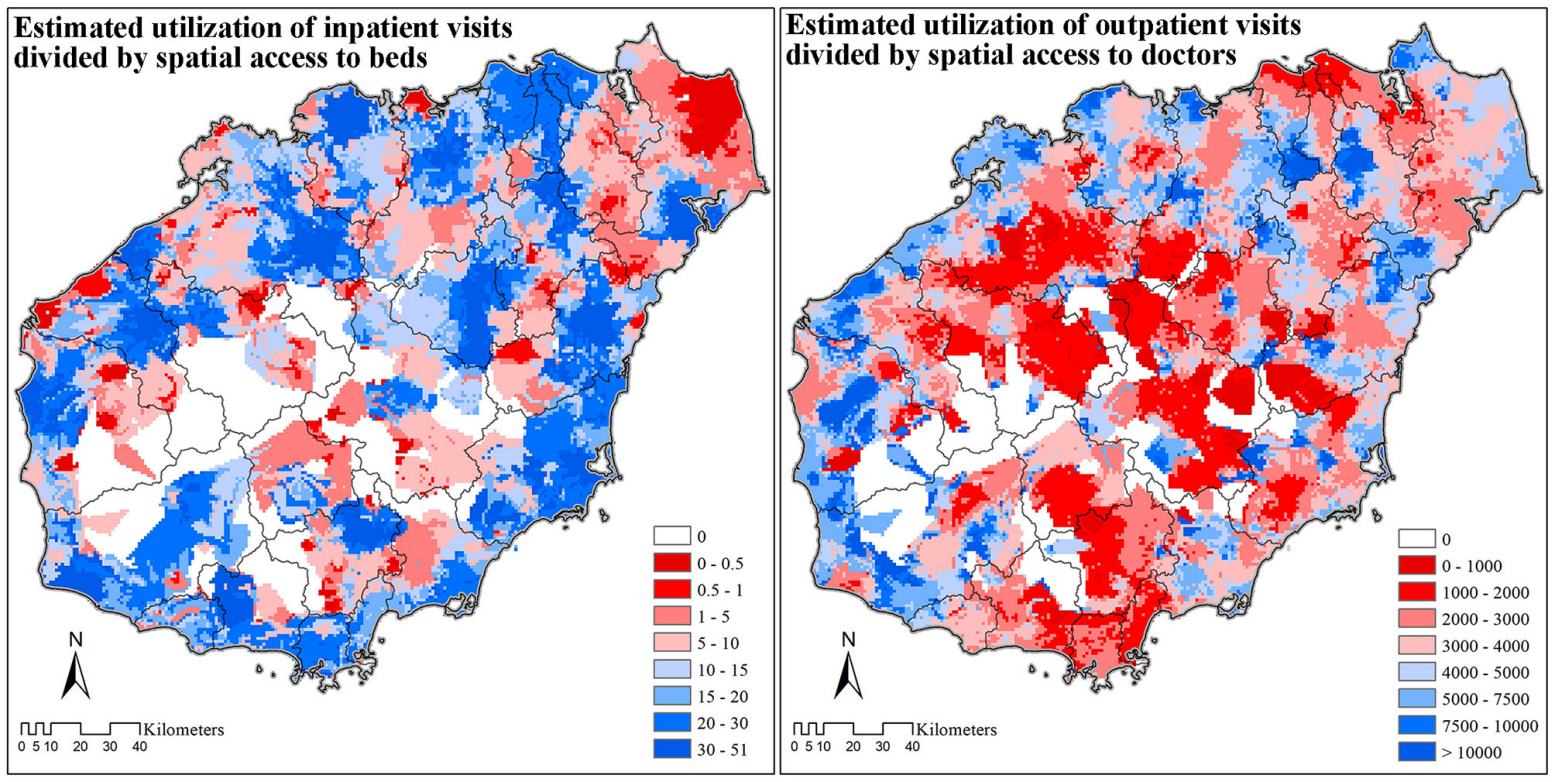
The efficiency of healthcare resources on Hainan Island (2018).

Results based on the EPHI index (Figures 3, 5) could also be used for resource allocation and policy making. By identifying regions according to local spatial access and patient spatial movements, specific assessment techniques can be carried out to provide key insights for resource allocation and policymaking. These four combinations are: (1) low spatial access with flow-out patients, (2) low spatial access with flow-in patients, (3) high spatial access with flow-out patients, and (4) high spatial access with flow-in patients. For areas with low spatial access and flow-out patients, more healthcare resources should be allocated to encourage patients to obtain services locally. While for areas with high spatial access and flow-in populations, the expansion of healthcare institutions should be restricted, and instead of large general hospitals, specialized hospitals treating complex diseases that local healthcare institutions cannot treat should be developed. Because the EPHI values are at regional level specific for each resident point, instead of for healthcare institutions and administrative units, the results can be used for both the allocation of healthcare resources in existing healthcare institutions, as well as for the investment of new healthcare institutions or enhancement of existing primary healthcare institutions.

Thus, the proposed index has great potential to provide guidance for government policy makers and future healthcare planning. In the case study carried out on Hainan Island, we tested the potential utility of this EPHI index.

The EPHI values show that bypass activities in both outpatient and inpatient care are common on Hainan. For both inpatient and outpatient needs, patients tend to travel from regions that are less developed with fewer healthcare resources (such as the central mountainous regions), to regions that are more highly developed with more healthcare resources (e.g., coasts and urban areas) to obtain medical care. These bypass activities have resulted in inefficient allocation of resources, with insufficient utilization of doctors in less-developed regions and over-utilization of doctors in coastal areas. Nevertheless, doctor resources in the two major urban centers along the coast, namely Haikou and Sanya cities, are still sufficient despite their large numbers of guest (flow-in) patients. One possible explanation is that the doctor resources in Haikou and Sanya are ample enough to serve the high inflow of patients from other areas across the island. Thus, it can be said that some doctor resources in Haikou and Sanya should be moved to other coastal areas to improve the general efficiency of doctor resources on Hainan Island.

One of the most challenging problems with the current situation on Hainan, as identified by the EPHI values, is that there is an unreasonable distribution of healthcare resources in terms of hospitals vs. primary healthcare institutions. The problem is more severe for inpatient visits since about 85% of inpatient beds are provided by the limited number of hospitals. This inevitably causes patients to bypass their more local healthcare institutions to seek inpatient care from hospitals. Patients increasingly choose to access higher level hospitals in China, because primary healthcare institutions are perceived as not trustworthy for safely addressing basic patient healthcare needs (27). This is because compared with hospitals, registered (assistant) doctors in primary healthcare institutions usually hold lower-level degrees and technical titles, with the majority of health professionals in primary healthcare institutions only having 2–3 years of medical education from technical secondary schools and only hold primary-level technical titles (58). The limited number of resources in primary healthcare institutions has further aggravated these perception difficulties.

Based on the current patterns of resource allocation and utilization revealed by our methods, and in light of the need to more efficiently utilize healthcare resources across Hainan Island, this study demonstrates the value of the EPHI for local governments. In particular, the Hainan provincial government seeks to provide timely access to healthcare at the local level according to its “Receiving Treatment at Local” policy (31). To do so, more efforts must be made to increase the primary healthcare institutional service capacities, as well as to study the exact reasons why local people's perceptions of these institutions do or do not align with their current services (e.g., both quality and type of services provided). With the improvement of both local healthcare capacity and quality, the bypass activity should reduce if local people's perceptions recognize these changes. At the same time, policies to guide rational patient flows should be examined in more detail.

Our study has several limitations that should be kept in mind. First, in comparison with other relevant research from Hainan Island (13), the proposed EPHI to simulate the spatial tendency for patient flow was generally based on the E2SFCA method, which has a strong assumption that the service catchment of healthcare institutions are fixed and only resident points within the catchment can enjoy the healthcare services, as well as provide patients, the guest patients have flow into these resident points to compete for healthcare resources allocated to local residents. The setting of catchment size and distance decay function would influence the results greatly. For further improvement of this index, other modifications of the 2SFCA method should be considered and individual-level medical records would be necessary to improve parameter setting. Second, patient morbidity was assumed to be the same across Hainan Island, but this likely differs based on social, demographic, and economic reasons in different regions and communities across the island. Thus, future studies should also assess the implications of differing patient morbidity on this type of analysis. Third, the EPHI value should be resulted from both the over/under-utilization of local residents and the competition of guest/bypass patients (resulted from the spatial tendencies in healthcare seeking), while only the spatial tendencies in healthcare seeking were discussed in this study under the background that unreasonable spatial tendencies in healthcare seeking is common and troublesome to the government in China. Nevertheless, the EPHI proposed for the first time in this article can provide good foundational knowledge for subsequent resource allocation research.

## Conclusion

Because the accurate assessment of both existing healthcare resources and their actual utilization are fundamentally crucial for effective allocation of limited healthcare resources, in this project, we proposed a new EPHI to measure the spatial tendency for healthcare seeking behavior in a more practical way. This index can be combined with spatial accessibility results to assess the utilization-supply regions to reveal healthcare competition and resource efficiency patterns. In China, patients are free to choose healthcare institutions for treatment, which increases the importance of modeling healthcare seeking behavior, including bypass activities. The proposed index was examined on Hainan Island, which is a relatively closed-loop system within and representative of China's broader healthcare economy. This EPHI index allowed us to identify patterns of both patient in-flow and patient out-flow, including the specific areas where each behavior predominates. By combining utilization data with resource allocation data, we could identify over-resourced areas and insufficiently-resourced areas, demonstrating the value of this index to provide guidance for local government planning. In future studies, the simulated results could be combined with social-economic parameters to assess influencing factors and test the effectiveness of specific policies.

## Data Availability Statement

The data analyzed in this study is subject to the following licenses/restrictions: Some data from this study are available from the Health Commission of Hainan Province, but restrictions limit their availability. These data were used under license for the current study, but are not publicly available. Data may be made available from the authors upon reasonable request to the corresponding author and with permission from the Health Commission of Hainan Province. Requests to access these datasets should be directed to panjie.jay@scu.edu.cn.

## Author Contributions

XW and JP designed the study and had full access to all the data involved in the study. XW performed the data analysis and wrote the first draft with supervision from JP and BS. CS and DW helped with study design. BS and DW helped revise the manuscript. All authors contributed to interpretation of data, revised the article critically for important intellectual content, and approved the final version of the manuscript. All authors have agreed to be accountable for all aspects of the work in ensuring that questions related to the accuracy or integrity of any part of the work are appropriately investigated and resolved.

## Funding

This study was supported by the National Natural Science Foundation of China (Grant Nos. 71874116, 42071379, and 72074163), National Social Science Fund of China (Grant No. 21ZDA104), Sichuan Science and Technology Program (Grant Nos. 2020YJ0117, 2021YFQ0060, and 22ZDYF0318), the Medical Science and Technology Project of Sichuan Provincial Health Commission (Grant No. 21PJ067), the Chengdu Federation of Social Sciences (Grant No. ZZ05), Fund for Introducing Talents of Sichuan University (YJ202157), Chongqing Science and Technology Bureau (cstc2020jscx-cylhX0001), the Sichuan University Interdisciplinary Postdoc Innovation Fund, and the Sichuan University Postdoc Research Foundation (Grant No. 2019SCU12013). No funder was involved in study design, data collection or analysis, decision to publish, or preparation of the manuscript.

## Conflict of Interest

The authors declare that the research was conducted in the absence of any commercial or financial relationships that could be construed as a potential conflict of interest.

## Publisher's Note

All claims expressed in this article are solely those of the authors and do not necessarily represent those of their affiliated organizations, or those of the publisher, the editors and the reviewers. Any product that may be evaluated in this article, or claim that may be made by its manufacturer, is not guaranteed or endorsed by the publisher.

## Abbreviations

EPHI: External Patient Healthcare Index
PPRs: Provider-to-population ratios
2SFCA: Two-Step Floating Catchment Area method
E2SFCA: Enhanced Two Step Floating Catchment Area method.
